# Ciprofloxacin salt and salt co-crystal with di­hydroxy­benzoic acids

**DOI:** 10.1107/S2056989022001177

**Published:** 2022-02-03

**Authors:** Yuda Prasetya Nugraha, Haruki Sugiyama, Hidehiro Uekusa

**Affiliations:** a Department of Pharmaceutics, School of Pharmacy, Bandung Institute of Technology, Bandung 40132, Indonesia; b Research and Education Center for Natural Sciences, Keio University, Hiyoshi, 4-1-1, Kohoku, Yokohama 223-8521, Japan; c Department of Life and Coordination-Complex Molecular Science, Institute for Molecular Science, Myodaiji, Okazaki 444-8787, Japan; d Department of Chemistry, Tokyo Institute of Technology, 2-12-1, Ookayama, Meguro, Tokyo 152-8551, Japan

**Keywords:** crystal structure, fluoro­quinolone, ciprofloxacin, di­hydroxy­benzoic acid, salt co-crystal, anti­biotic

## Abstract

The crystal structure of two multi-component crystals of ciprofloxacin [systematic name: 1-cyclo­propyl-6-fluoro-4-oxo-7-(piperazin-1-yl)quinoline-3-carb­­oxy­lic acid], a fluoro­quinolone anti­biotic, namely, ciprofloxacin 2,6-di­hydroxy­benzoate salt, C_17_H_19_FN_3_O_3_^+^·C_7_H_5_O_4_^−^, (I), and ciprofloxacin hydro­chloride–3,5-di­hydroxy­benzoic–water (1/1/1), C_17_H_19_FN_3_O_3_^+^·Cl^−^·C_7_H_6_O_4_·H_2_O, (II), were determined. In (I) and (II), the ciprofloxacin cations are connected *via* head-to-tail N—H⋯O hydrogen bonding. Both structures show an alternating layered arrangement between ciprofloxacin and di­hydroxy­benzoic acid.

## Abstract 2

The crystal structure of a ciprofloxacin salt with 2,6-di­hydroxy­benzoic acid and a ciprofloxacin hydro­chloride salt co-crystal with 3,5-di­hydroxy­benzoic acid are reported.

## Chemical context

The design and exploration of multi-component crystals of active pharmaceutical ingredients (APIs) have gained increasing inter­est over recent decades. The formation of multi-component crystals, *i.e.* salts and co-crystals through a crystal-engineering approach has been continuously demonstrated as a versatile tool to improve the physicochemical properties of APIs (Kavanagh *et al.*, 2019 ▸; Putra & Uekusa, 2020 ▸; Thakur & Thakuria, 2020 ▸). Recently, the co-crystallization of salt APIs or salt co-crystal formation has been increasingly studied. Salt co-crystallization has been utilized to suppress hydrate formation of salt APIs (Nugraha & Uekusa, 2018 ▸; Fujito *et al.*, 2021 ▸). As a part of our study of salt co-crystals of APIs, we investigated multi-component crystals of ciprofloxacin. Ciprofloxacin is a Biopharmaceutics Classification System (BCS) class IV fluoro­quinolone anti­biotic that is widely used therapeutically as the free base and the hydro­chloride salt (Olivera *et al.*, 2011 ▸). 
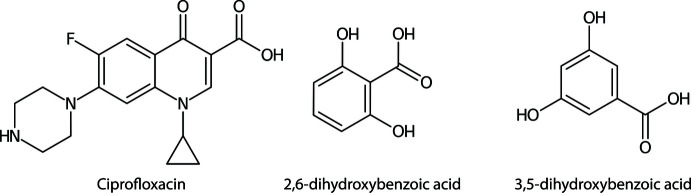


## Structural commentary

**Figure 1:**
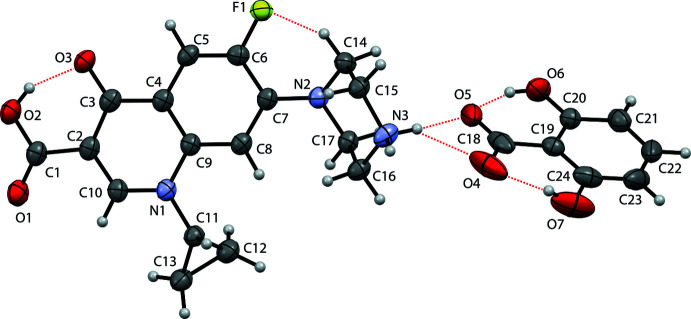
Displacement ellipsoid (50% probability level) drawing with the atomic labelling scheme for compound (I) showing the hydrogen bonds within the selected asymmetric unit.

Compound (I) was obtained as an anion-exchange product between ciprofloxacin hydro­chloride and 2,6-di­hydro­benzoic acid in solution. 2,6-Di­hydroxy­benzoic acid (2,6HBA) is a relatively strong carb­oxy­lic acid with a p*K*_a_ of 1.30 (Gdaniec *et al.*, 1994 ▸; Habibi-yangjeh *et al.*, 2005 ▸). Compound (I) crystallizes in the monoclinic space group *P*2_1_/*c*. The asymmetric unit consists of one ciprofloxacin cation and one 2,6HBA anion (Fig. 1 ▸). The C—O distances of the ciprofloxacin carb­oxy­lic group *i.e.*, 1.218 (3) and 1.325 (3) Å indicate that it exists as the neutral carb­oxy­lic form. However, in 2,6HBA, the C–O distances are very similar *i.e.*, 1.263 (4) and 1.267 (3) Å due to resonance stabilization in the carboxyl­ate anion (Childs *et al.*, 2007 ▸; Aakeröy *et al.*, 2006 ▸). As a result, the piperazinyl group of ciprofloxacin is protonated. Therefore, compound (I) is a salt. The formation of a salt is well-predicted by the p*K*_a_ rule (Cruz-Cabeza, 2012 ▸). The p*K*_a_ of ciprofloxacin are 6.18 and 8.73 for the carb­oxy­lic acid and the piperazinyl ring, respectively (Sun *et al.*, 2002 ▸). Therefore, salt formation is expected because the Δp*K*_a_ between the piperazinyl ring of ciprofloxacin and the carb­oxy­lic acid of 2,6HBA is greater than 4. Similar behaviour is observed in the salicylate salt of ciprofloxacin (Surov *et al.*, 2019 ▸; Nugrahani *et al.*, 2020 ▸).

**Figure 2:**
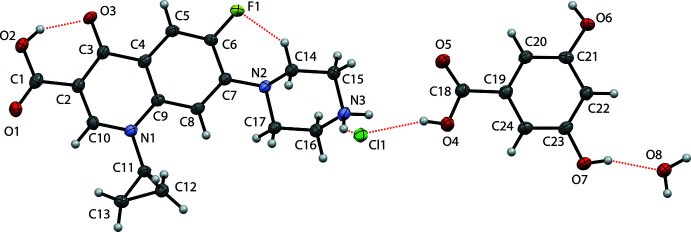
Displacement ellipsoid (50% probability level) drawing with the atomic labelling scheme for compound (II) showing the hydrogen bonds within the selected asymmetric unit.

Compound (II) crystallizes in the non-centrosymmetric *P*1 space group despite the lack of a chiral centre. The asymmetric unit comprises one ciprofloxacin cation, one chloride anion and one 3,5HBA mol­ecule, as shown in Fig. 2 ▸. In addition, one water mol­ecule is incorporated into the crystal lattice. An anion-exchange reaction during crystallization did not occur in this system. Compared to 2,6HBA, the coformer is a weaker acid with a p*K*_a_ of 4.04 (Habibi-yangjeh *et al.*, 2005 ▸). Contrary to the previous structures, the coformer exists as a neutral mol­ecule in the crystal. The carb­oxy­lic C18—O4 and C18—O5 distances of 2,6HBA are 1.320 (4) and 1.216 (4) Å, respectively, confirming its neutral state. Additionally, the carb­oxy­lic C1—O1 and C1—O2 distances of ciprofloxacin, *i.e.* 1.227 (4) and 1.314 (4) Å, respectively, also confirm the neutral state of this moiety. On the other hand, the piperazinyl group is protonated. Hence, compound (II) is a salt co-crystal monohydrate of ciprofloxacin.

**Figure 3:**
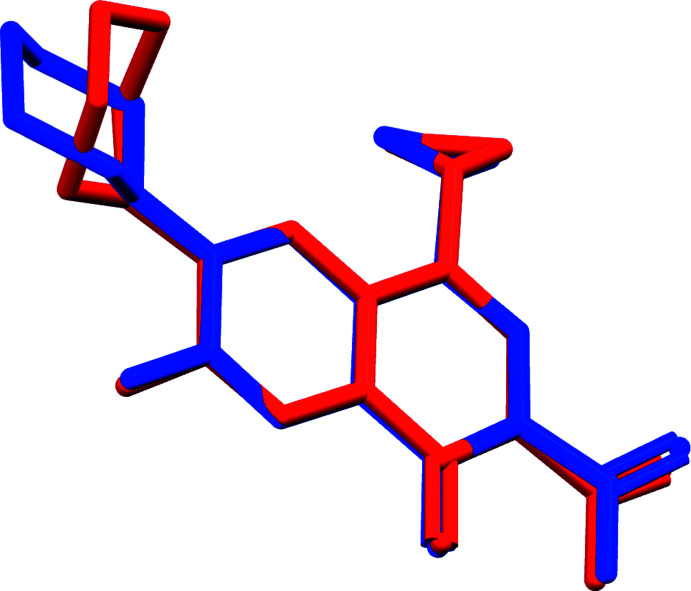
Mol­ecular overlay of ciprofloxacin cation in compounds (I) (red) and (II) (blue). Hydrogen atoms are omitted for clarity.

Compounds (I) and (II) exhibit similar conformations, as shown in Fig. 3 ▸. The mol­ecular conformation of the ciprofloxacin mol­ecule is governed by intra­molecular O2—H2⋯O3 and C14—H14*A*⋯F1 hydrogen bonding (Tables 1 ▸ and 2 ▸). In both structures, the piperazinium ring exhibits a chair conformation. The main difference is the relative orientation between the piperazinium moiety and the quinolone ring. The C7—N2—C14—C15 torsion angles are 97.0 (2) and −167.8 (2)°, respectively, for compounds (I) and (II).

## Supra­molecular features

**Figure 4:**
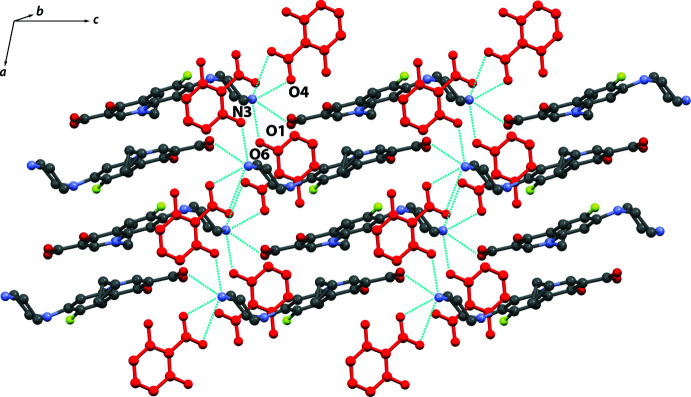
Inter­molecular hydrogen-bonding motifs in (I) showing infinite chains along the *a-*axis direction formed by ciprofloxacin and 2,6HBA (red). Hydrogen atoms are omitted for clarity.

**Figure 5:**
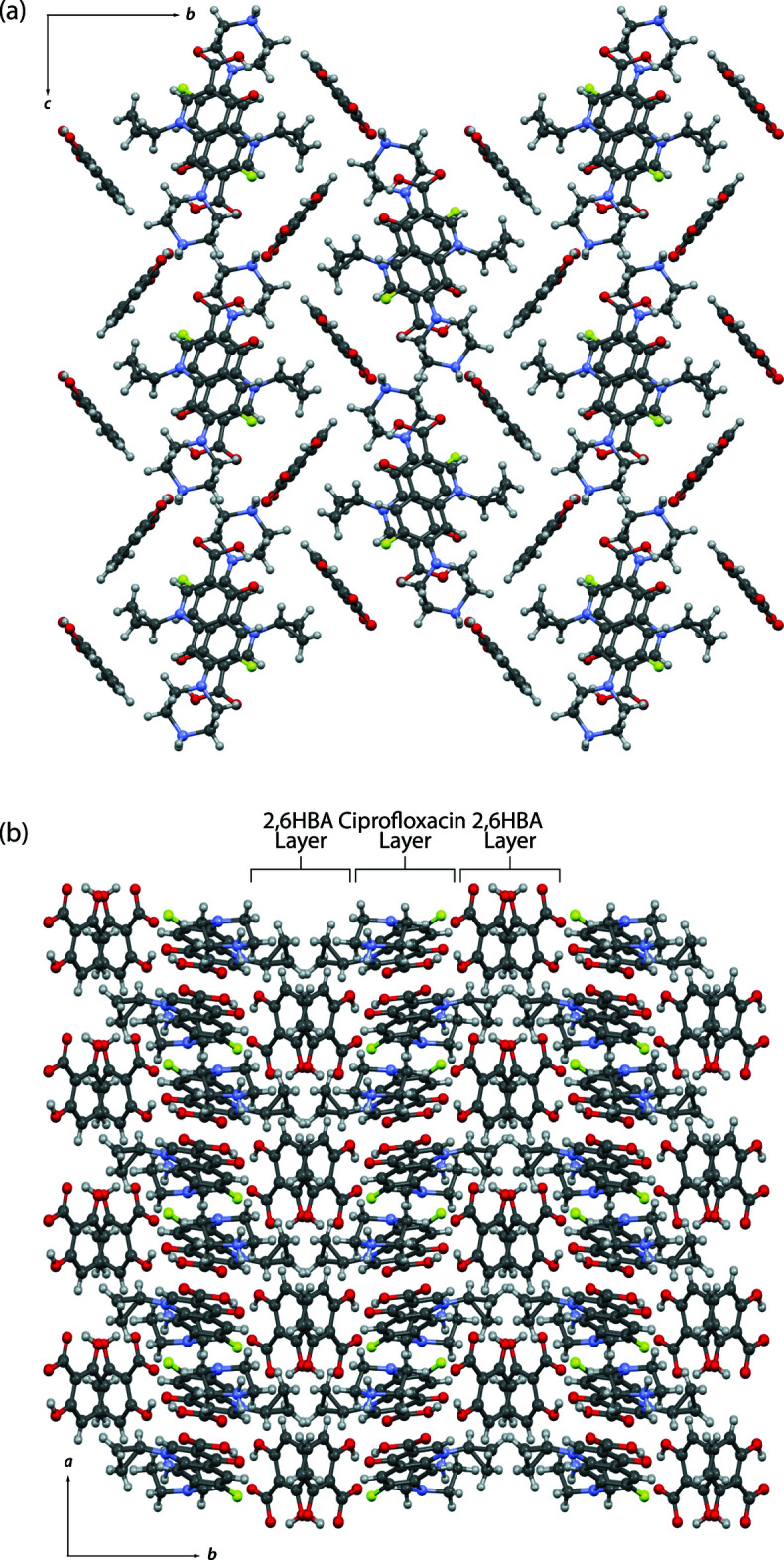
Packing motifs of (I) viewed along (*a*) the *a* axis and (*b*) the *c* axis highlighting the alternating layers of ciprofloxacin and the coformer.

In compound (I), the carboxyl­ate anion of 2,6HBA acts as a hydrogen-bond donor for intra­molecular hydrogen bonds involving two hydroxyl groups, namely O6—H6⋯O5 and O7—H7⋯O4. The protonated nitro­gen atom N3 of the piperazinyl ring is involved in the formation of trifurcated hydrogen bonds with O4, O5, and O6 of the coformer. These charge-assisted hydrogen bonds, *i.e.* N3—H3*B*⋯O4, N3—H3*B*⋯O5, and N3—H3*A*⋯O6, form an infinite chain structure along the *a*-axis direction (Table 1 ▸, Fig. 4 ▸). The chains are connected to the adjacent ciprofloxacin mol­ecule through head-to-tail N3—H3*A*⋯O1 hydrogen bonding. The crystal packing of (I) is shown in Fig. 5 ▸. Along the *a*-axis, centrosymmetric pairs of ciprofloxacin mol­ecules are stacked by *π*–*π* inter­actions. The distance between the centroids of symmetry-related C4–C9 rings is 3.4986 (11) Å. This arrangement leads to the formation of a columnar packing arrangement. Inter­estingly, a similar packing feature was observed in the 1.75 hydrate of ciprofloxacin salicylate (Nugrahani *et al.*, 2020 ▸). In addition, compound (I) shows a layered structure with alternating ciprofloxacin and 2,6HBA layers along the *b* axis.

**Figure 6:**
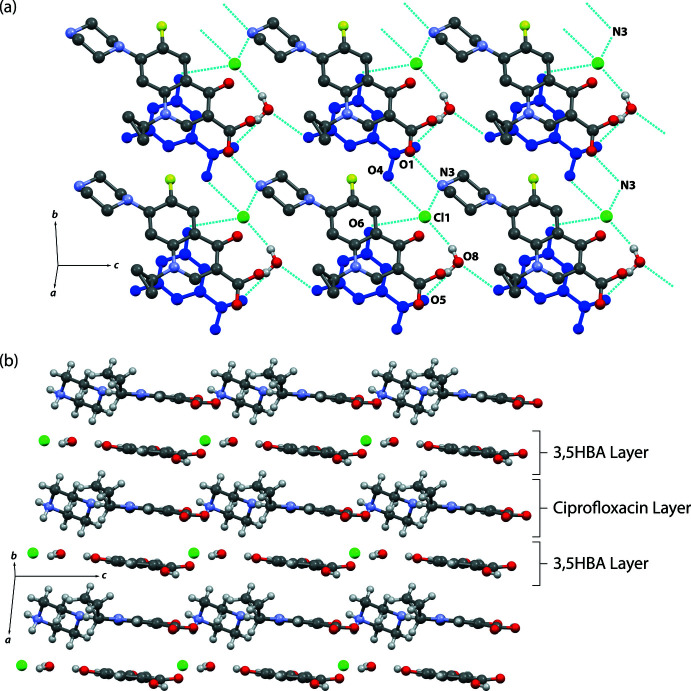
Inter­molecular hydrogen-bonding motifs in (II) highlighting the role of the chloride ion and water mol­ecule in bridging ciprofloxacin and 3,5HBA (blue). Hydrogen atoms are omitted for clarity. (*b*) The crystal packing of (II) showing the alternating layered arrangement.

The supra­molecular features of compound (II) are similar to those observed in compound (I). Ciprofloxacin cations are inter­connected through head-to-tail N3—H3*A*⋯O1 hydrogen bonds (Table 2 ▸), forming an infinite chain arrangement. The chloride ion and water mol­ecule are involved in an extensive hydrogen-bond network bridging ciprofloxacin and 3,5HBA (Fig. 6 ▸ *a*). Inter­estingly, compound (II) also shows a layered arrangement of ciprofloxacin and the coformer (Fig. 6 ▸ *b*).

## Database survey

Several crystal structures of ciprofloxacin salts with benzoic acid derivatives have been reported, including salts with salicylic acid (Surov *et al.*, 2019 ▸; Nagalapalli & Yaga Bheem, 2014 ▸; CSD refcode family DOFWUT), 4-hy­droxy­benzoic acid (Surov *et al.*, 2020 ▸; CSD refcode PUNMUJ), 4-amino­benzoic acid (Surov *et al.*, 2020 ▸; CSD refcode PUNMIX) and gallic acid (Surov *et al.*, 2020 ▸; CSD refcode PUNMOD). A search for salt co-crystals of ciprofloxacin hydro­chloride yielded one reported crystal structure, a co-crystal of ciprofloxacin hydro­chloride with 4-hy­droxy­benzoic acid (Martínez-Alejo *et al.*, 2014 ▸; CSD refcode XOHTUL). Compound (II) was also disclosed in a patent without any structural information (Rojas *et al.*, 2016 ▸).

## Synthesis and crystallization

Single crystals of (I) and (II) were obtained by preparing a saturated solution of equimolar ciprofloxacin hydro­chloride and the respective coformer in methanol/water (1:1) at room temperature. The saturated solution was allowed to slowly evaporate at room temperature. A suitable single crystal was selected and measured for structure determination.

## Refinement

Crystal data, data collection and structure refinement details are summarized in Table 3 ▸. All non-hydrogen atoms were refined anisotropically. All hydrogen atoms were refined using a riding model and their displacement parameters (*U*_iso_) were fixed to 1.2*U*_eq_ of the parent carbon or nitro­gen atom and 1.5*U*_eq_ for hydroxyl groups.

## Supplementary Material

Crystal structure: contains datablock(s) I, II. DOI: 10.1107/S2056989022001177/dx2042sup1.cif

Structure factors: contains datablock(s) I. DOI: 10.1107/S2056989022001177/dx2042Isup2.hkl

Structure factors: contains datablock(s) II. DOI: 10.1107/S2056989022001177/dx2042IIsup3.hkl

Supporting information file. DOI: 10.1107/S2056989022001177/dx2042Isup4.cml

CCDC references: 2098049, 2098403

Additional supporting information:  crystallographic information; 3D view; checkCIF report

## Figures and Tables

**Table 1:** Hydrogen-bond geometry (Å, °) for (I)

*D*—H⋯*A*	*D*—H	H⋯*A*	*D*⋯*A*	*D*—H⋯*A*
O2—H2⋯O3	0.84	1.73	2.512 (2)	155
N3—H3*A*⋯O1^i^	0.91	2.38	2.977 (2)	123
N3—H3*A*⋯O6	0.91	2.09	2.890 (2)	146
N3—H3*B*⋯O4^ii^	0.91	2.18	2.897 (3)	136
N3—H3*B*⋯O5^ii^	0.91	2.24	3.090 (3)	155
C11—H11⋯O3^iii^	1.00	2.46	3.239 (3)	134
C12—H12*A*⋯O4^iv^	0.99	2.54	3.374 (3)	141
C13—H13*A*⋯O7^v^	0.99	2.51	3.193 (3)	126
C14—H14*A*⋯F1	0.99	2.13	2.831 (2)	126
C15—H15*B*⋯O1^iii^	0.99	2.33	3.282 (3)	161
C17—H17*A*⋯O5^ii^	0.99	2.60	3.408 (3)	139
O6—H6⋯O5	0.84	1.77	2.520 (3)	148
O7—H7⋯O4	0.84	1.85	2.508 (4)	134
C21—H21⋯O4^ii^	0.95	2.54	3.488 (3)	178

Symmetry codes: (i) 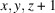 ; (ii) 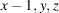 ; (iii) 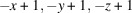 ; (iv) 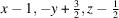 ; (v) 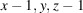 .

**Table 2:** Hydrogen-bond geometry (Å, °) for (II)

*D*—H⋯*A*	*D*—H	H⋯*A*	*D*⋯*A*	*D*—H⋯*A*
O2—H2⋯O3	0.84	1.78	2.551 (3)	152
N3—H3*A*⋯O1^i^	0.91	1.75	2.652 (3)	172
N3—H3*B*⋯Cl1	0.91	2.30	3.106 (3)	148
C10—H10⋯F1^ii^	0.95	2.46	3.158 (4)	130
C12—H12*B*⋯O7^iii^	0.99	2.47	3.435 (4)	166
C14—H14*B*⋯F1	0.99	2.27	2.927 (3)	123
C16—H16*B*⋯Cl1^iv^	0.99	2.78	3.609 (3)	142
O4—H4⋯Cl1	0.84	2.28	3.082 (2)	160
O6—H6⋯Cl1^v^	0.84	2.40	3.232 (2)	170
O7—H7⋯O8	0.84	1.96	2.769 (3)	161
O8—H8*A*⋯Cl1^i^	0.88 (6)	2.51 (6)	3.362 (3)	164 (4)
O8—H8*B*⋯O5^vi^	0.82 (6)	2.05 (6)	2.865 (4)	170 (5)

Symmetry codes: (i) 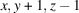 ; (ii) 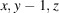 ; (iii) 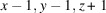 ; (iv) 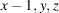 ; (v) 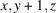 ; (vi) 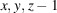 .

**Table 3:** Experimental details

	(I)	(II)
Crystal data		
Chemical formula	C_17_H_19_FN_3_O_3_^+^·C_7_H_5_O_4_^−^	C_17_H_19_FN_3_O_3_^+^·C_7_H_6_O_4_·Cl^−^·H_2_O
*M*_r_	485.46	539.93
Crystal system, space group	Monoclinic, *P*2_1_/*c*	Triclinic, *P*1
Temperature (K)	93	93
*a*, *b*, *c* (Å)	7.9722 (5), 21.2705 (11), 13.0860 (7)	7.2165 (2), 8.8298 (4), 10.1184 (3)
α, β, γ (°)	90, 101.805 (6), 90	92.997 (3), 95.219 (2), 111.557 (4)
*V* (Å^3^)	2172.1 (2)	594.60 (4)
*Z*	4	1
Radiation type	Cu *K*α	Cu *K*α
μ (mm^−1^)	0.98	2.00
Crystal size (mm)	0.23 × 0.05 × 0.04	0.28 × 0.2 × 0.05

Data collection		
Diffractometer	XtaLAB Synergy R, DW system, HyPix	XtaLAB Synergy R, DW system, HyPix
Absorption correction	Multi-scan (*CrysAlis PRO*; Rigaku OD, 2020 ▸)	Multi-scan (*CrysAlis PRO*; Rigaku OD, 2020 ▸)
*T*_min_, *T*_max_	0.919, 1.000	0.839, 1.000
No. of measured, independent and observed reflections	15936, 4378, 3601 (?)	16358, 4420, 4323 [*I* > 2σ(*I*)]
*R*_int_	0.038	0.035
(sin θ/λ)_max_ (Å^−1^)	0.630	0.625

Refinement		
*R*[*F* ^2^ > 2σ(*F* ^2^)], *wR*(*F* ^2^), *S*	0.053, 0.139, 1.04	0.034, 0.094, 1.12
No. of reflections	4378	4420
No. of parameters	319	344
No. of restraints	0	3
H-atom treatment	H-atom parameters constrained	H atoms treated by a mixture of independent and constrained refinement
Δρ_max_, Δρ_min_ (e Å^−3^)	0.34, −0.41	0.25, −0.47
Absolute structure	–	Flack *x* determined using 1889 quotients [(*I* ^+^)−(*I* ^−^)]/[(*I* ^+^)+(*I* ^−^)] (Parsons *et al.*, 2013 ▸)
Absolute structure parameter	–	0.011 (7)

Computer programs: *CrysAlis PRO* (Rigaku OD, 2020 ▸), *SHELXT2018/2* (Sheldrick, 2015*a* ▸), *SHELXL2018/3* (Sheldrick, 2015*b* ▸), *OLEX2* (Dolomanov *et al.*, 2009 ▸) and *Mercury* (Macrae *et al.*, 2020 ▸).

## supplementary crystallographic information

### 4-(3-Carboxy-1-cyclopropyl-6-fluoro-4-oxo-1,4-dihydroquinolin-7-yl)piperazin-1-ium 2,6-dihydroxybenzoate (I) Crystal data

**Table:**

C_17_H_19_FN_3_O_3_^+^·C_7_H_5_O_4_^−^	*F*(000) = 1016
*M**_r_* = 485.46	*D*_x_ = 1.485 Mg m^−^^3^
Monoclinic, *P*2_1_/*c*	Cu *K*α radiation, λ = 1.54184 Å
*a* = 7.9722 (5) Å	Cell parameters from 4777 reflections
*b* = 21.2705 (11) Å	θ = 4.0–72.0°
*c* = 13.0860 (7) Å	µ = 0.98 mm^−^^1^
β = 101.805 (6)°	*T* = 93 K
*V* = 2172.1 (2) Å^3^	Block, colourless
*Z* = 4	0.23 × 0.05 × 0.04 mm

### 4-(3-Carboxy-1-cyclopropyl-6-fluoro-4-oxo-1,4-dihydroquinolin-7-yl)piperazin-1-ium 2,6-dihydroxybenzoate (I) Data collection

**Table:**

XtaLAB Synergy R, DW system, HyPix diffractometer	15936 measured reflections
Radiation source: Rotating-anode X-ray tube, Rigaku XtaLAB Synergy-R	4378 independent reflections
Mirror monochromator	*R*_int_ = 0.038
Detector resolution: 10.0000 pixels mm^-1^	θ_max_ = 76.3°, θ_min_ = 4.0°
ω scans	*h* = −9→9
Absorption correction: multi-scan (CrysAlisPro; Rigaku OD, 2020)	*k* = −17→26
*T*_min_ = 0.919, *T*_max_ = 1.000	*l* = −15→16

### 4-(3-Carboxy-1-cyclopropyl-6-fluoro-4-oxo-1,4-dihydroquinolin-7-yl)piperazin-1-ium 2,6-dihydroxybenzoate (I) Refinement

**Table:**

Refinement on *F*^2^	Primary atom site location: dual
Least-squares matrix: full	Hydrogen site location: inferred from neighbouring sites
*R*[*F*^2^ > 2σ(*F*^2^)] = 0.053	H-atom parameters constrained
*wR*(*F*^2^) = 0.139	*w* = 1/[σ^2^(*F*_o_^2^) + (0.062*P*)^2^ + 1.4432*P*] where *P* = (*F*_o_^2^ + 2*F*_c_^2^)/3
*S* = 1.03	(Δ/σ)_max_ = 0.001
4378 reflections	Δρ_max_ = 0.34 e Å^−^^3^
319 parameters	Δρ_min_ = −0.41 e Å^−^^3^
0 restraints	

### 4-(3-Carboxy-1-cyclopropyl-6-fluoro-4-oxo-1,4-dihydroquinolin-7-yl)piperazin-1-ium 2,6-dihydroxybenzoate (I) Special details

**Table:**

Geometry. All esds (except the esd in the dihedral angle between two l.s. planes) are estimated using the full covariance matrix. The cell esds are taken into account individually in the estimation of esds in distances, angles and torsion angles; correlations between esds in cell parameters are only used when they are defined by crystal symmetry. An approximate (isotropic) treatment of cell esds is used for estimating esds involving l.s. planes.

### 4-(3-Carboxy-1-cyclopropyl-6-fluoro-4-oxo-1,4-dihydroquinolin-7-yl)piperazin-1-ium 2,6-dihydroxybenzoate (I) Fractional atomic coordinates and isotropic or equivalent isotropic displacement parameters (Å^2^)

**Table:**

	*x*	*y*	*z*	*U*_iso_*/*U*_eq_	
F1	0.05295 (16)	0.41493 (5)	0.67770 (9)	0.0377 (3)	
O1	0.4436 (2)	0.54707 (8)	0.16888 (11)	0.0432 (4)	
O2	0.3833 (2)	0.44645 (7)	0.18852 (11)	0.0402 (4)	
H2	0.347742	0.424196	0.232696	0.060*	
O3	0.28730 (19)	0.41034 (7)	0.35014 (11)	0.0362 (3)	
N1	0.3471 (2)	0.59129 (8)	0.46044 (12)	0.0314 (4)	
N2	0.0934 (2)	0.53783 (8)	0.75639 (13)	0.0328 (4)	
N3	0.2572 (3)	0.58568 (8)	0.95730 (14)	0.0395 (4)	
H3A	0.356800	0.595565	1.002153	0.047*	
H3B	0.169225	0.594851	0.989447	0.047*	
C1	0.4001 (3)	0.50538 (10)	0.22173 (15)	0.0354 (5)	
C2	0.3602 (3)	0.51643 (10)	0.32635 (15)	0.0327 (4)	
C3	0.3006 (2)	0.46647 (10)	0.38290 (15)	0.0326 (4)	
C4	0.2566 (2)	0.48427 (10)	0.48093 (15)	0.0316 (4)	
C5	0.1856 (3)	0.44006 (9)	0.54010 (15)	0.0327 (4)	
H5	0.172151	0.397574	0.517532	0.039*	
C6	0.1363 (3)	0.45781 (9)	0.62931 (15)	0.0321 (4)	
C7	0.1580 (2)	0.51970 (9)	0.67053 (15)	0.0313 (4)	
C8	0.2342 (2)	0.56281 (9)	0.61296 (15)	0.0314 (4)	
H8	0.256130	0.604419	0.638611	0.038*	
C9	0.2786 (2)	0.54598 (9)	0.51851 (15)	0.0303 (4)	
C10	0.3838 (3)	0.57567 (10)	0.36771 (15)	0.0329 (4)	
H10	0.428110	0.607231	0.329187	0.040*	
C11	0.3814 (3)	0.65431 (9)	0.50290 (16)	0.0338 (4)	
H11	0.471754	0.657418	0.568093	0.041*	
C12	0.2333 (3)	0.69888 (10)	0.49555 (18)	0.0418 (5)	
H12A	0.232822	0.727421	0.555245	0.050*	
H12B	0.118861	0.684208	0.459028	0.050*	
C13	0.3671 (3)	0.70946 (10)	0.43140 (17)	0.0407 (5)	
H13A	0.334605	0.701423	0.355393	0.049*	
H13B	0.448510	0.744615	0.451562	0.049*	
C14	0.1041 (3)	0.49851 (10)	0.84970 (16)	0.0357 (5)	
H14A	0.115360	0.453863	0.830791	0.043*	
H14B	−0.002908	0.502959	0.876377	0.043*	
C15	0.2556 (3)	0.51692 (9)	0.93462 (16)	0.0340 (4)	
H15A	0.250824	0.493237	0.999094	0.041*	
H15B	0.363113	0.505307	0.912586	0.041*	
C16	0.2411 (3)	0.62529 (10)	0.86112 (16)	0.0364 (5)	
H16A	0.344474	0.620481	0.830902	0.044*	
H16B	0.229875	0.670139	0.878811	0.044*	
C17	0.0831 (3)	0.60408 (10)	0.78271 (15)	0.0327 (4)	
H17A	−0.020076	0.611142	0.812303	0.039*	
H17B	0.071566	0.629659	0.718442	0.039*	
O4	1.1188 (2)	0.66620 (11)	1.0984 (2)	0.0830 (8)	
O5	0.8981 (3)	0.62083 (8)	0.99400 (18)	0.0683 (6)	
O6	0.5971 (2)	0.63810 (8)	1.02051 (13)	0.0476 (4)	
H6	0.675991	0.625422	0.991803	0.071*	
O7	1.0721 (3)	0.74339 (12)	1.23479 (19)	0.0809 (8)	
H7	1.128472	0.712831	1.218955	0.121*	
C18	0.9592 (3)	0.65706 (12)	1.0688 (2)	0.0531 (7)	
C19	0.8411 (3)	0.68834 (9)	1.12632 (16)	0.0336 (4)	
C20	0.6639 (3)	0.67641 (10)	1.10169 (16)	0.0332 (4)	
C21	0.5540 (3)	0.70341 (11)	1.1582 (2)	0.0447 (5)	
H21	0.434867	0.694228	1.142088	0.054*	
C22	0.6208 (4)	0.74400 (12)	1.2386 (2)	0.0593 (8)	
H22	0.545515	0.763145	1.277098	0.071*	
C23	0.7929 (5)	0.75756 (13)	1.2647 (2)	0.0625 (8)	
H23	0.835717	0.785656	1.320376	0.075*	
C24	0.9025 (3)	0.72996 (12)	1.20927 (18)	0.0475 (6)	

### 4-(3-Carboxy-1-cyclopropyl-6-fluoro-4-oxo-1,4-dihydroquinolin-7-yl)piperazin-1-ium 2,6-dihydroxybenzoate (I) Atomic displacement parameters (Å^2^)

**Table:**

	*U*^11^	*U*^22^	*U*^33^	*U*^12^	*U*^13^	*U*^23^
F1	0.0444 (7)	0.0350 (6)	0.0334 (6)	−0.0065 (5)	0.0070 (5)	−0.0004 (5)
O1	0.0555 (10)	0.0466 (9)	0.0264 (7)	0.0049 (7)	0.0058 (7)	0.0000 (7)
O2	0.0470 (9)	0.0437 (9)	0.0280 (7)	0.0011 (7)	0.0030 (6)	−0.0068 (6)
O3	0.0384 (8)	0.0360 (8)	0.0312 (7)	0.0019 (6)	0.0003 (6)	−0.0065 (6)
N1	0.0347 (9)	0.0338 (9)	0.0239 (8)	0.0014 (7)	0.0017 (7)	0.0003 (6)
N2	0.0370 (9)	0.0345 (9)	0.0254 (8)	−0.0019 (7)	0.0030 (7)	−0.0007 (7)
N3	0.0477 (10)	0.0353 (9)	0.0290 (9)	0.0021 (8)	−0.0074 (8)	−0.0025 (7)
C1	0.0360 (11)	0.0418 (11)	0.0255 (10)	0.0036 (9)	−0.0008 (8)	−0.0026 (9)
C2	0.0300 (10)	0.0390 (11)	0.0260 (9)	0.0034 (8)	−0.0014 (8)	−0.0031 (8)
C3	0.0288 (9)	0.0364 (10)	0.0288 (10)	0.0040 (8)	−0.0033 (8)	−0.0031 (8)
C4	0.0290 (9)	0.0370 (10)	0.0254 (9)	0.0016 (8)	−0.0022 (7)	−0.0011 (8)
C5	0.0331 (10)	0.0313 (10)	0.0298 (10)	0.0020 (8)	−0.0024 (8)	−0.0035 (8)
C6	0.0304 (10)	0.0340 (10)	0.0293 (10)	−0.0024 (8)	−0.0001 (8)	0.0016 (8)
C7	0.0300 (9)	0.0360 (10)	0.0253 (9)	0.0006 (8)	−0.0002 (7)	−0.0010 (8)
C8	0.0308 (10)	0.0337 (10)	0.0265 (9)	0.0011 (8)	−0.0017 (7)	−0.0016 (8)
C9	0.0297 (10)	0.0345 (10)	0.0242 (9)	0.0027 (8)	−0.0001 (7)	0.0005 (8)
C10	0.0332 (10)	0.0387 (11)	0.0249 (9)	0.0031 (8)	0.0014 (8)	0.0011 (8)
C11	0.0398 (11)	0.0325 (10)	0.0282 (10)	−0.0005 (8)	0.0053 (8)	−0.0009 (8)
C12	0.0457 (12)	0.0372 (11)	0.0420 (12)	0.0049 (9)	0.0074 (10)	−0.0010 (9)
C13	0.0538 (13)	0.0345 (11)	0.0328 (11)	0.0025 (9)	0.0067 (9)	0.0015 (9)
C14	0.0406 (11)	0.0380 (11)	0.0275 (10)	−0.0058 (9)	0.0047 (8)	0.0008 (8)
C15	0.0385 (11)	0.0330 (10)	0.0283 (10)	0.0013 (8)	0.0018 (8)	−0.0005 (8)
C16	0.0410 (11)	0.0326 (10)	0.0321 (10)	0.0006 (8)	−0.0011 (9)	−0.0021 (8)
C17	0.0335 (10)	0.0359 (10)	0.0269 (9)	0.0020 (8)	0.0017 (8)	−0.0010 (8)
O4	0.0390 (10)	0.0790 (14)	0.140 (2)	0.0173 (9)	0.0391 (12)	0.0559 (15)
O5	0.1042 (17)	0.0351 (9)	0.0862 (15)	0.0002 (10)	0.0675 (13)	−0.0015 (10)
O6	0.0513 (10)	0.0531 (10)	0.0356 (8)	−0.0164 (8)	0.0027 (7)	−0.0101 (7)
O7	0.0613 (12)	0.0922 (16)	0.0708 (14)	−0.0414 (11)	−0.0296 (11)	0.0281 (13)
C18	0.0488 (14)	0.0374 (13)	0.082 (2)	0.0124 (10)	0.0340 (14)	0.0259 (13)
C19	0.0320 (10)	0.0335 (10)	0.0344 (10)	−0.0010 (8)	0.0048 (8)	0.0063 (8)
C20	0.0342 (10)	0.0347 (10)	0.0296 (10)	−0.0019 (8)	0.0036 (8)	0.0007 (8)
C21	0.0394 (12)	0.0431 (12)	0.0547 (14)	0.0024 (10)	0.0172 (10)	0.0040 (11)
C22	0.096 (2)	0.0364 (13)	0.0590 (16)	−0.0030 (13)	0.0477 (16)	−0.0036 (11)
C23	0.108 (2)	0.0474 (14)	0.0343 (12)	−0.0330 (15)	0.0198 (14)	−0.0087 (11)
C24	0.0525 (14)	0.0496 (13)	0.0341 (11)	−0.0194 (11)	−0.0056 (10)	0.0104 (10)

### 4-(3-Carboxy-1-cyclopropyl-6-fluoro-4-oxo-1,4-dihydroquinolin-7-yl)piperazin-1-ium 2,6-dihydroxybenzoate (I) Geometric parameters (Å, º)

**Table:**

F1—C6	1.359 (2)	C12—H12A	0.9900
O1—C1	1.218 (3)	C12—H12B	0.9900
O2—H2	0.8400	C12—C13	1.503 (3)
O2—C1	1.325 (3)	C13—H13A	0.9900
O3—C3	1.265 (2)	C13—H13B	0.9900
N1—C9	1.405 (3)	C14—H14A	0.9900
N1—C10	1.347 (3)	C14—H14B	0.9900
N1—C11	1.455 (3)	C14—C15	1.516 (3)
N2—C7	1.383 (3)	C15—H15A	0.9900
N2—C14	1.468 (3)	C15—H15B	0.9900
N2—C17	1.457 (3)	C16—H16A	0.9900
N3—H3A	0.9100	C16—H16B	0.9900
N3—H3B	0.9100	C16—C17	1.521 (3)
N3—C15	1.492 (3)	C17—H17A	0.9900
N3—C16	1.498 (3)	C17—H17B	0.9900
C1—C2	1.486 (3)	O4—C18	1.267 (3)
C2—C3	1.431 (3)	O5—C18	1.263 (4)
C2—C10	1.369 (3)	O6—H6	0.8400
C3—C4	1.448 (3)	O6—C20	1.358 (3)
C4—C5	1.408 (3)	O7—H7	0.8400
C4—C9	1.400 (3)	O7—C24	1.355 (3)
C5—H5	0.9500	C18—C19	1.479 (3)
C5—C6	1.359 (3)	C19—C20	1.406 (3)
C6—C7	1.420 (3)	C19—C24	1.409 (3)
C7—C8	1.402 (3)	C20—C21	1.382 (3)
C8—H8	0.9500	C21—H21	0.9500
C8—C9	1.400 (3)	C21—C22	1.381 (4)
C10—H10	0.9500	C22—H22	0.9500
C11—H11	1.0000	C22—C23	1.375 (5)
C11—C12	1.501 (3)	C23—H23	0.9500
C11—C13	1.490 (3)	C23—C24	1.376 (4)
			
C1—O2—H2	109.5	C13—C12—H12B	117.8
C9—N1—C11	119.24 (16)	C11—C13—C12	60.21 (15)
C10—N1—C9	119.88 (17)	C11—C13—H13A	117.8
C10—N1—C11	120.86 (17)	C11—C13—H13B	117.8
C7—N2—C14	123.30 (17)	C12—C13—H13A	117.8
C7—N2—C17	120.67 (17)	C12—C13—H13B	117.8
C17—N2—C14	110.55 (16)	H13A—C13—H13B	114.9
H3A—N3—H3B	107.8	N2—C14—H14A	109.4
C15—N3—H3A	109.0	N2—C14—H14B	109.4
C15—N3—H3B	109.0	N2—C14—C15	111.36 (17)
C15—N3—C16	112.86 (16)	H14A—C14—H14B	108.0
C16—N3—H3A	109.0	C15—C14—H14A	109.4
C16—N3—H3B	109.0	C15—C14—H14B	109.4
O1—C1—O2	121.63 (19)	N3—C15—C14	111.86 (17)
O1—C1—C2	123.19 (19)	N3—C15—H15A	109.2
O2—C1—C2	115.18 (19)	N3—C15—H15B	109.2
C3—C2—C1	121.03 (18)	C14—C15—H15A	109.2
C10—C2—C1	118.14 (19)	C14—C15—H15B	109.2
C10—C2—C3	120.83 (19)	H15A—C15—H15B	107.9
O3—C3—C2	122.61 (19)	N3—C16—H16A	110.0
O3—C3—C4	121.90 (19)	N3—C16—H16B	110.0
C2—C3—C4	115.48 (18)	N3—C16—C17	108.50 (17)
C5—C4—C3	120.65 (18)	H16A—C16—H16B	108.4
C9—C4—C3	121.38 (19)	C17—C16—H16A	110.0
C9—C4—C5	117.95 (18)	C17—C16—H16B	110.0
C4—C5—H5	119.8	N2—C17—C16	111.57 (16)
C6—C5—C4	120.40 (19)	N2—C17—H17A	109.3
C6—C5—H5	119.8	N2—C17—H17B	109.3
F1—C6—C5	117.95 (18)	C16—C17—H17A	109.3
F1—C6—C7	118.58 (18)	C16—C17—H17B	109.3
C5—C6—C7	123.34 (19)	H17A—C17—H17B	108.0
N2—C7—C6	122.04 (18)	C20—O6—H6	109.5
N2—C7—C8	121.89 (18)	C24—O7—H7	109.5
C8—C7—C6	115.79 (18)	O4—C18—C19	118.7 (3)
C7—C8—H8	119.2	O5—C18—O4	122.3 (3)
C9—C8—C7	121.53 (19)	O5—C18—C19	119.0 (2)
C9—C8—H8	119.2	C20—C19—C18	121.1 (2)
C4—C9—N1	119.20 (18)	C20—C19—C24	117.7 (2)
C4—C9—C8	120.89 (19)	C24—C19—C18	121.2 (2)
C8—C9—N1	119.91 (18)	O6—C20—C19	120.12 (19)
N1—C10—C2	123.07 (19)	O6—C20—C21	118.6 (2)
N1—C10—H10	118.5	C21—C20—C19	121.3 (2)
C2—C10—H10	118.5	C20—C21—H21	120.7
N1—C11—H11	115.6	C22—C21—C20	118.7 (2)
N1—C11—C12	118.27 (18)	C22—C21—H21	120.7
N1—C11—C13	120.07 (17)	C21—C22—H22	119.0
C12—C11—H11	115.6	C23—C22—C21	122.0 (2)
C13—C11—H11	115.6	C23—C22—H22	119.0
C13—C11—C12	60.32 (15)	C22—C23—H23	120.4
C11—C12—H12A	117.8	C22—C23—C24	119.3 (2)
C11—C12—H12B	117.8	C24—C23—H23	120.4
C11—C12—C13	59.47 (14)	O7—C24—C19	119.6 (3)
H12A—C12—H12B	115.0	O7—C24—C23	119.3 (3)
C13—C12—H12A	117.8	C23—C24—C19	121.1 (2)
			
F1—C6—C7—N2	−1.6 (3)	C9—C4—C5—C6	1.8 (3)
F1—C6—C7—C8	−175.68 (16)	C10—N1—C9—C4	2.8 (3)
O1—C1—C2—C3	176.50 (19)	C10—N1—C9—C8	−177.48 (18)
O1—C1—C2—C10	−4.2 (3)	C10—N1—C11—C12	102.8 (2)
O2—C1—C2—C3	−2.7 (3)	C10—N1—C11—C13	32.6 (3)
O2—C1—C2—C10	176.59 (18)	C10—C2—C3—O3	−175.52 (18)
O3—C3—C4—C5	−4.5 (3)	C10—C2—C3—C4	4.3 (3)
O3—C3—C4—C9	177.25 (17)	C11—N1—C9—C4	−175.72 (17)
N1—C11—C12—C13	−110.4 (2)	C11—N1—C9—C8	4.0 (3)
N1—C11—C13—C12	107.5 (2)	C11—N1—C10—C2	177.41 (18)
N2—C7—C8—C9	−171.43 (18)	C14—N2—C7—C6	42.6 (3)
N2—C14—C15—N3	52.0 (2)	C14—N2—C7—C8	−143.72 (19)
N3—C16—C17—N2	−58.5 (2)	C14—N2—C17—C16	61.2 (2)
C1—C2—C3—O3	3.8 (3)	C15—N3—C16—C17	53.4 (2)
C1—C2—C3—C4	−176.44 (17)	C16—N3—C15—C14	−51.4 (2)
C1—C2—C10—N1	178.08 (18)	C17—N2—C7—C6	−166.08 (18)
C2—C3—C4—C5	175.74 (17)	C17—N2—C7—C8	7.6 (3)
C2—C3—C4—C9	−2.5 (3)	C17—N2—C14—C15	−56.9 (2)
C3—C2—C10—N1	−2.6 (3)	O4—C18—C19—C20	−175.4 (2)
C3—C4—C5—C6	−176.56 (18)	O4—C18—C19—C24	3.0 (3)
C3—C4—C9—N1	−0.9 (3)	O5—C18—C19—C20	2.6 (3)
C3—C4—C9—C8	179.39 (18)	O5—C18—C19—C24	−179.0 (2)
C4—C5—C6—F1	173.48 (16)	O6—C20—C21—C22	−177.9 (2)
C4—C5—C6—C7	−2.5 (3)	C18—C19—C20—O6	−3.4 (3)
C5—C4—C9—N1	−179.21 (17)	C18—C19—C20—C21	177.1 (2)
C5—C4—C9—C8	1.1 (3)	C18—C19—C24—O7	2.3 (3)
C5—C6—C7—N2	174.32 (18)	C18—C19—C24—C23	−178.0 (2)
C5—C6—C7—C8	0.2 (3)	C19—C20—C21—C22	1.6 (3)
C6—C7—C8—C9	2.7 (3)	C20—C19—C24—O7	−179.2 (2)
C7—N2—C14—C15	97.0 (2)	C20—C19—C24—C23	0.5 (3)
C7—N2—C17—C16	−93.5 (2)	C20—C21—C22—C23	−0.9 (4)
C7—C8—C9—N1	176.92 (17)	C21—C22—C23—C24	0.0 (4)
C7—C8—C9—C4	−3.4 (3)	C22—C23—C24—O7	179.9 (2)
C9—N1—C10—C2	−1.1 (3)	C22—C23—C24—C19	0.2 (4)
C9—N1—C11—C12	−78.7 (2)	C24—C19—C20—O6	178.08 (19)
C9—N1—C11—C13	−148.89 (19)	C24—C19—C20—C21	−1.4 (3)

### 4-(3-Carboxy-1-cyclopropyl-6-fluoro-4-oxo-1,4-dihydroquinolin-7-yl)piperazin-1-ium 2,6-dihydroxybenzoate (I) Hydrogen-bond geometry (Å, º)

**Table:**

*D*—H···*A*	*D*—H	H···*A*	*D*···*A*	*D*—H···*A*
O2—H2···O3	0.84	1.73	2.512 (2)	155
N3—H3*A*···O1^i^	0.91	2.38	2.977 (2)	123
N3—H3*A*···O6	0.91	2.09	2.890 (2)	146
N3—H3*B*···O4^ii^	0.91	2.18	2.897 (3)	136
N3—H3*B*···O5^ii^	0.91	2.24	3.090 (3)	155
C11—H11···O3^iii^	1.00	2.46	3.239 (3)	134
C12—H12*A*···O4^iv^	0.99	2.54	3.374 (3)	141
C13—H13*A*···O7^v^	0.99	2.51	3.193 (3)	126
C14—H14*A*···F1	0.99	2.13	2.831 (2)	126
C15—H15*B*···O1^iii^	0.99	2.33	3.282 (3)	161
C17—H17*A*···O5^ii^	0.99	2.60	3.408 (3)	139
O6—H6···O5	0.84	1.77	2.520 (3)	148
O7—H7···O4	0.84	1.85	2.508 (4)	134
C21—H21···O4^ii^	0.95	2.54	3.488 (3)	178

Symmetry codes: (i) *x*, *y*, *z*+1; (ii) *x*−1, *y*, *z*; (iii) −*x*+1, −*y*+1, −*z*+1; (iv) *x*−1, −*y*+3/2, *z*−1/2; (v) *x*−1, *y*, *z*−1.

### 4-(3-Carboxy-1-cyclopropyl-6-fluoro-4-oxo-1,4-dihydroquinolin-7-yl)piperazin-1-ium chloride–3,5-hydroxybenzoic acid–water (1/1/1) (II) Crystal data

**Table:**

C_17_H_19_FN_3_O_3_^+^·C_7_H_6_O_4_·Cl^−^·H_2_O	*Z* = 1
*M**_r_* = 539.93	*F*(000) = 282
Triclinic, *P*1	*D*_x_ = 1.508 Mg m^−^^3^
*a* = 7.2165 (2) Å	Cu *K*α radiation, λ = 1.54184 Å
*b* = 8.8298 (4) Å	Cell parameters from 10041 reflections
*c* = 10.1184 (3) Å	θ = 4.4–75.8°
α = 92.997 (3)°	µ = 2.00 mm^−^^1^
β = 95.219 (2)°	*T* = 93 K
γ = 111.557 (4)°	Block, colourless
*V* = 594.60 (4) Å^3^	0.28 × 0.2 × 0.05 mm

### 4-(3-Carboxy-1-cyclopropyl-6-fluoro-4-oxo-1,4-dihydroquinolin-7-yl)piperazin-1-ium chloride–3,5-hydroxybenzoic acid–water (1/1/1) (II) Data collection

**Table:**

XtaLAB Synergy R, DW system, HyPix diffractometer	4420 independent reflections
Radiation source: Rotating-anode X-ray tube, Rigaku XtaLAB Synergy-R	4323 reflections with *I* > 2σ(*I*)
Mirror monochromator	*R*_int_ = 0.035
Detector resolution: 10.0000 pixels mm^-1^	θ_max_ = 74.5°, θ_min_ = 4.4°
ω scans	*h* = −9→9
Absorption correction: multi-scan (CrysAlisPro; Rigaku OD, 2020)	*k* = −10→11
*T*_min_ = 0.839, *T*_max_ = 1.000	*l* = −12→12
16358 measured reflections	

### 4-(3-Carboxy-1-cyclopropyl-6-fluoro-4-oxo-1,4-dihydroquinolin-7-yl)piperazin-1-ium chloride–3,5-hydroxybenzoic acid–water (1/1/1) (II) Refinement

**Table:**

Refinement on *F*^2^	Hydrogen site location: mixed
Least-squares matrix: full	H atoms treated by a mixture of independent and constrained refinement
*R*[*F*^2^ > 2σ(*F*^2^)] = 0.034	*w* = 1/[σ^2^(*F*_o_^2^) + (0.0589*P*)^2^ + 0.0709*P*] where *P* = (*F*_o_^2^ + 2*F*_c_^2^)/3
*wR*(*F*^2^) = 0.094	(Δ/σ)_max_ < 0.001
*S* = 1.12	Δρ_max_ = 0.25 e Å^−^^3^
4420 reflections	Δρ_min_ = −0.47 e Å^−^^3^
344 parameters	Absolute structure: Flack *x* determined using 1889 quotients [(*I*^+^)-(*I*^-^)]/[(*I*^+^)+(*I*^-^)] (Parsons *et al.*, 2013)
3 restraints	Absolute structure parameter: 0.011 (7)
Primary atom site location: dual	

### 4-(3-Carboxy-1-cyclopropyl-6-fluoro-4-oxo-1,4-dihydroquinolin-7-yl)piperazin-1-ium chloride–3,5-hydroxybenzoic acid–water (1/1/1) (II) Special details

**Table:**

Geometry. All esds (except the esd in the dihedral angle between two l.s. planes) are estimated using the full covariance matrix. The cell esds are taken into account individually in the estimation of esds in distances, angles and torsion angles; correlations between esds in cell parameters are only used when they are defined by crystal symmetry. An approximate (isotropic) treatment of cell esds is used for estimating esds involving l.s. planes.

### 4-(3-Carboxy-1-cyclopropyl-6-fluoro-4-oxo-1,4-dihydroquinolin-7-yl)piperazin-1-ium chloride–3,5-hydroxybenzoic acid–water (1/1/1) (II) Fractional atomic coordinates and isotropic or equivalent isotropic displacement parameters (Å^2^)

**Table:**

	*x*	*y*	*z*	*U*_iso_*/*U*_eq_	
Cl1	0.85144 (9)	0.46863 (8)	0.40663 (7)	0.02533 (17)	
F1	0.4599 (3)	0.5185 (2)	0.98137 (17)	0.0273 (4)	
O1	0.3224 (3)	−0.3703 (3)	1.2978 (2)	0.0284 (5)	
O2	0.3642 (4)	−0.1499 (3)	1.4315 (2)	0.0294 (5)	
H2	0.379572	−0.051835	1.423817	0.044*	
O3	0.3972 (3)	0.1143 (3)	1.3272 (2)	0.0280 (5)	
N1	0.2840 (4)	−0.1445 (3)	0.9603 (2)	0.0213 (5)	
N2	0.3796 (4)	0.3457 (3)	0.7334 (2)	0.0210 (5)	
N3	0.4407 (4)	0.4661 (3)	0.4778 (2)	0.0233 (5)	
H3A	0.412202	0.525266	0.413562	0.028*	
H3B	0.528778	0.423848	0.448534	0.028*	
C1	0.3370 (4)	−0.2274 (4)	1.3126 (3)	0.0249 (6)	
C2	0.3276 (4)	−0.1342 (4)	1.1970 (3)	0.0222 (6)	
C3	0.3655 (4)	0.0366 (4)	1.2138 (3)	0.0226 (6)	
C4	0.3687 (4)	0.1155 (4)	1.0907 (3)	0.0220 (6)	
C5	0.4158 (4)	0.2850 (4)	1.0937 (3)	0.0227 (6)	
H5	0.444144	0.349621	1.176480	0.027*	
C6	0.4203 (4)	0.3556 (4)	0.9773 (3)	0.0214 (6)	
C7	0.3828 (4)	0.2667 (4)	0.8503 (3)	0.0202 (6)	
C8	0.3389 (4)	0.1003 (4)	0.8483 (3)	0.0212 (6)	
H8	0.314618	0.036841	0.765286	0.025*	
C9	0.3297 (4)	0.0241 (4)	0.9661 (3)	0.0204 (6)	
C10	0.2882 (4)	−0.2168 (4)	1.0730 (3)	0.0218 (6)	
H10	0.262685	−0.330555	1.066643	0.026*	
C11	0.2568 (4)	−0.2397 (4)	0.8324 (3)	0.0219 (6)	
H11	0.381927	−0.228926	0.791979	0.026*	
C12	0.0740 (5)	−0.2667 (4)	0.7366 (3)	0.0257 (6)	
H12A	0.087678	−0.270948	0.640183	0.031*	
H12B	−0.021768	−0.217677	0.763308	0.031*	
C13	0.0926 (5)	−0.4047 (4)	0.8124 (3)	0.0259 (6)	
H13A	0.007825	−0.440061	0.885206	0.031*	
H13B	0.117236	−0.493315	0.762121	0.031*	
C14	0.5686 (4)	0.4771 (4)	0.7139 (3)	0.0231 (6)	
H14A	0.667670	0.429259	0.691921	0.028*	
H14B	0.623427	0.549862	0.797431	0.028*	
C15	0.5351 (5)	0.5757 (4)	0.6022 (3)	0.0253 (6)	
H15A	0.447094	0.632955	0.628102	0.030*	
H15B	0.665144	0.659131	0.586453	0.030*	
C16	0.2536 (5)	0.3306 (4)	0.5001 (3)	0.0240 (6)	
H16A	0.198514	0.256713	0.417103	0.029*	
H16B	0.152583	0.375766	0.523119	0.029*	
C17	0.2940 (4)	0.2351 (4)	0.6112 (3)	0.0221 (6)	
H17A	0.167365	0.147006	0.626540	0.027*	
H17B	0.388812	0.184215	0.586309	0.027*	
O4	0.8307 (4)	0.7243 (3)	0.2157 (2)	0.0292 (5)	
H4	0.845149	0.674900	0.282288	0.044*	
O5	0.8281 (4)	0.9108 (3)	0.3721 (2)	0.0338 (5)	
O6	0.9253 (4)	1.4048 (3)	0.1019 (2)	0.0282 (5)	
H6	0.922943	1.426566	0.183393	0.042*	
O7	0.7685 (4)	0.9471 (3)	−0.2173 (2)	0.0282 (5)	
H7	0.769716	1.019031	−0.268691	0.042*	
C18	0.8310 (5)	0.8689 (4)	0.2561 (3)	0.0245 (6)	
C19	0.8373 (4)	0.9759 (4)	0.1448 (3)	0.0233 (6)	
C20	0.8753 (4)	1.1395 (4)	0.1782 (3)	0.0230 (6)	
H20	0.894255	1.180995	0.269053	0.028*	
C21	0.8855 (4)	1.2429 (4)	0.0771 (3)	0.0230 (6)	
C22	0.8531 (4)	1.1805 (4)	−0.0565 (3)	0.0229 (6)	
H22	0.860643	1.250739	−0.125512	0.028*	
C23	0.8099 (4)	1.0150 (4)	−0.0877 (3)	0.0231 (6)	
C24	0.8048 (4)	0.9107 (4)	0.0123 (3)	0.0243 (6)	
H24	0.779908	0.798399	−0.009350	0.029*	
O8	0.7775 (4)	1.1327 (3)	−0.4306 (2)	0.0309 (5)	
H8A	0.799 (7)	1.230 (7)	−0.457 (5)	0.046*	
H8B	0.779 (7)	1.068 (7)	−0.492 (5)	0.046*	

### 4-(3-Carboxy-1-cyclopropyl-6-fluoro-4-oxo-1,4-dihydroquinolin-7-yl)piperazin-1-ium chloride–3,5-hydroxybenzoic acid–water (1/1/1) (II) Atomic displacement parameters (Å^2^)

**Table:**

	*U*^11^	*U*^22^	*U*^33^	*U*^12^	*U*^13^	*U*^23^
Cl1	0.0315 (3)	0.0267 (3)	0.0219 (3)	0.0151 (3)	0.0052 (2)	0.0029 (2)
F1	0.0445 (10)	0.0200 (9)	0.0203 (8)	0.0152 (8)	0.0057 (7)	−0.0001 (7)
O1	0.0376 (12)	0.0253 (12)	0.0242 (11)	0.0142 (9)	0.0011 (9)	0.0058 (9)
O2	0.0419 (13)	0.0289 (12)	0.0176 (10)	0.0132 (10)	0.0032 (9)	0.0048 (8)
O3	0.0426 (13)	0.0294 (12)	0.0144 (9)	0.0160 (10)	0.0048 (9)	0.0003 (8)
N1	0.0270 (12)	0.0220 (13)	0.0168 (12)	0.0117 (10)	0.0024 (9)	−0.0002 (9)
N2	0.0255 (12)	0.0232 (13)	0.0144 (11)	0.0097 (10)	0.0009 (9)	0.0008 (9)
N3	0.0303 (12)	0.0286 (13)	0.0173 (11)	0.0174 (10)	0.0047 (9)	0.0046 (10)
C1	0.0243 (14)	0.0325 (18)	0.0202 (14)	0.0131 (12)	0.0027 (11)	0.0050 (12)
C2	0.0238 (13)	0.0266 (15)	0.0179 (13)	0.0108 (11)	0.0036 (10)	0.0041 (12)
C3	0.0262 (14)	0.0272 (15)	0.0175 (14)	0.0131 (12)	0.0044 (11)	0.0038 (11)
C4	0.0234 (13)	0.0268 (15)	0.0177 (13)	0.0117 (11)	0.0035 (10)	0.0007 (11)
C5	0.0282 (14)	0.0232 (15)	0.0182 (13)	0.0117 (12)	0.0036 (10)	−0.0011 (11)
C6	0.0267 (14)	0.0176 (13)	0.0206 (14)	0.0094 (11)	0.0032 (11)	−0.0006 (11)
C7	0.0222 (13)	0.0228 (15)	0.0171 (13)	0.0105 (11)	0.0018 (10)	0.0016 (11)
C8	0.0239 (13)	0.0230 (15)	0.0167 (13)	0.0095 (11)	0.0021 (10)	−0.0023 (11)
C9	0.0210 (13)	0.0217 (14)	0.0188 (14)	0.0084 (10)	0.0030 (10)	0.0005 (11)
C10	0.0242 (13)	0.0215 (15)	0.0225 (14)	0.0112 (11)	0.0033 (11)	0.0050 (11)
C11	0.0291 (14)	0.0213 (14)	0.0173 (13)	0.0121 (12)	0.0024 (11)	−0.0009 (11)
C12	0.0317 (15)	0.0243 (15)	0.0213 (13)	0.0117 (12)	−0.0005 (12)	0.0006 (11)
C13	0.0329 (15)	0.0211 (15)	0.0242 (14)	0.0110 (12)	0.0038 (11)	−0.0004 (11)
C14	0.0281 (14)	0.0233 (15)	0.0183 (13)	0.0103 (12)	0.0021 (11)	0.0011 (11)
C15	0.0335 (15)	0.0240 (15)	0.0198 (14)	0.0116 (12)	0.0050 (11)	0.0040 (11)
C16	0.0291 (15)	0.0241 (15)	0.0192 (13)	0.0111 (12)	0.0006 (11)	0.0012 (11)
C17	0.0264 (14)	0.0242 (16)	0.0174 (13)	0.0121 (11)	0.0006 (10)	0.0006 (11)
O4	0.0432 (13)	0.0270 (12)	0.0240 (11)	0.0194 (10)	0.0077 (9)	0.0056 (9)
O5	0.0540 (14)	0.0318 (13)	0.0216 (11)	0.0224 (11)	0.0074 (10)	0.0025 (9)
O6	0.0423 (12)	0.0214 (11)	0.0220 (10)	0.0133 (9)	0.0046 (9)	−0.0007 (8)
O7	0.0408 (12)	0.0264 (12)	0.0186 (10)	0.0140 (10)	0.0046 (9)	−0.0003 (9)
C18	0.0269 (14)	0.0271 (16)	0.0230 (14)	0.0137 (12)	0.0048 (11)	0.0025 (12)
C19	0.0240 (13)	0.0265 (16)	0.0216 (14)	0.0116 (11)	0.0039 (11)	0.0017 (12)
C20	0.0254 (13)	0.0255 (15)	0.0196 (13)	0.0117 (11)	0.0029 (11)	−0.0003 (11)
C21	0.0244 (13)	0.0205 (14)	0.0243 (14)	0.0092 (11)	0.0023 (11)	−0.0019 (11)
C22	0.0249 (14)	0.0247 (15)	0.0215 (14)	0.0115 (11)	0.0045 (11)	0.0035 (12)
C23	0.0240 (13)	0.0281 (16)	0.0187 (14)	0.0119 (12)	0.0023 (11)	−0.0010 (12)
C24	0.0261 (14)	0.0259 (15)	0.0232 (15)	0.0122 (12)	0.0058 (11)	−0.0007 (12)
O8	0.0438 (13)	0.0286 (13)	0.0212 (11)	0.0146 (10)	0.0044 (9)	0.0017 (9)

### 4-(3-Carboxy-1-cyclopropyl-6-fluoro-4-oxo-1,4-dihydroquinolin-7-yl)piperazin-1-ium chloride–3,5-hydroxybenzoic acid–water (1/1/1) (II) Geometric parameters (Å, º)

**Table:**

F1—C6	1.357 (4)	C12—C13	1.513 (4)
O1—C1	1.227 (4)	C13—H13A	0.9900
O2—H2	0.8400	C13—H13B	0.9900
O2—C1	1.314 (4)	C14—H14A	0.9900
O3—C3	1.263 (4)	C14—H14B	0.9900
N1—C9	1.397 (4)	C14—C15	1.517 (4)
N1—C10	1.339 (4)	C15—H15A	0.9900
N1—C11	1.463 (4)	C15—H15B	0.9900
N2—C7	1.407 (4)	C16—H16A	0.9900
N2—C14	1.468 (4)	C16—H16B	0.9900
N2—C17	1.467 (4)	C16—C17	1.510 (4)
N3—H3A	0.9100	C17—H17A	0.9900
N3—H3B	0.9100	C17—H17B	0.9900
N3—C15	1.489 (4)	O4—H4	0.8400
N3—C16	1.484 (4)	O4—C18	1.320 (4)
C1—C2	1.475 (4)	O5—C18	1.216 (4)
C2—C3	1.428 (4)	O6—H6	0.8400
C2—C10	1.369 (4)	O6—C21	1.356 (4)
C3—C4	1.457 (4)	O7—H7	0.8400
C4—C5	1.406 (4)	O7—C23	1.372 (4)
C4—C9	1.407 (4)	C18—C19	1.501 (4)
C5—H5	0.9500	C19—C20	1.385 (4)
C5—C6	1.358 (4)	C19—C24	1.395 (4)
C6—C7	1.419 (4)	C20—H20	0.9500
C7—C8	1.384 (4)	C20—C21	1.395 (4)
C8—H8	0.9500	C21—C22	1.399 (4)
C8—C9	1.394 (4)	C22—H22	0.9500
C10—H10	0.9500	C22—C23	1.389 (5)
C11—H11	1.0000	C23—C24	1.398 (4)
C11—C12	1.499 (4)	C24—H24	0.9500
C11—C13	1.492 (4)	O8—H8A	0.88 (6)
C12—H12A	0.9900	O8—H8B	0.82 (6)
C12—H12B	0.9900		
			
C1—O2—H2	109.5	C11—C13—C12	59.9 (2)
C9—N1—C11	120.6 (2)	C11—C13—H13A	117.8
C10—N1—C9	119.9 (3)	C11—C13—H13B	117.8
C10—N1—C11	119.0 (3)	C12—C13—H13A	117.8
C7—N2—C14	115.7 (2)	C12—C13—H13B	117.8
C7—N2—C17	114.7 (2)	H13A—C13—H13B	114.9
C17—N2—C14	111.0 (2)	N2—C14—H14A	109.5
H3A—N3—H3B	108.0	N2—C14—H14B	109.5
C15—N3—H3A	109.3	N2—C14—C15	110.6 (2)
C15—N3—H3B	109.3	H14A—C14—H14B	108.1
C16—N3—H3A	109.3	C15—C14—H14A	109.5
C16—N3—H3B	109.3	C15—C14—H14B	109.5
C16—N3—C15	111.4 (2)	N3—C15—C14	110.2 (2)
O1—C1—O2	121.8 (3)	N3—C15—H15A	109.6
O1—C1—C2	121.2 (3)	N3—C15—H15B	109.6
O2—C1—C2	117.0 (3)	C14—C15—H15A	109.6
C3—C2—C1	121.3 (3)	C14—C15—H15B	109.6
C10—C2—C1	117.3 (3)	H15A—C15—H15B	108.1
C10—C2—C3	121.4 (3)	N3—C16—H16A	109.5
O3—C3—C2	122.5 (3)	N3—C16—H16B	109.5
O3—C3—C4	122.3 (3)	N3—C16—C17	110.7 (2)
C2—C3—C4	115.2 (3)	H16A—C16—H16B	108.1
C5—C4—C3	120.8 (3)	C17—C16—H16A	109.5
C5—C4—C9	118.6 (3)	C17—C16—H16B	109.5
C9—C4—C3	120.6 (3)	N2—C17—C16	109.4 (2)
C4—C5—H5	120.3	N2—C17—H17A	109.8
C6—C5—C4	119.5 (3)	N2—C17—H17B	109.8
C6—C5—H5	120.3	C16—C17—H17A	109.8
F1—C6—C5	119.0 (3)	C16—C17—H17B	109.8
F1—C6—C7	117.8 (2)	H17A—C17—H17B	108.2
C5—C6—C7	123.2 (3)	C18—O4—H4	109.5
N2—C7—C6	120.4 (3)	C21—O6—H6	109.5
C8—C7—N2	122.6 (3)	C23—O7—H7	109.5
C8—C7—C6	116.9 (3)	O4—C18—C19	113.1 (3)
C7—C8—H8	119.4	O5—C18—O4	123.1 (3)
C7—C8—C9	121.1 (3)	O5—C18—C19	123.8 (3)
C9—C8—H8	119.4	C20—C19—C18	117.9 (3)
N1—C9—C4	119.8 (2)	C20—C19—C24	121.7 (3)
C8—C9—N1	119.6 (3)	C24—C19—C18	120.4 (3)
C8—C9—C4	120.7 (3)	C19—C20—H20	120.3
N1—C10—C2	123.0 (3)	C19—C20—C21	119.3 (3)
N1—C10—H10	118.5	C21—C20—H20	120.3
C2—C10—H10	118.5	O6—C21—C20	122.8 (3)
N1—C11—H11	116.2	O6—C21—C22	117.1 (3)
N1—C11—C12	118.9 (3)	C20—C21—C22	120.1 (3)
N1—C11—C13	117.2 (2)	C21—C22—H22	120.2
C12—C11—H11	116.2	C23—C22—C21	119.6 (3)
C13—C11—H11	116.2	C23—C22—H22	120.2
C13—C11—C12	60.8 (2)	O7—C23—C22	121.6 (3)
C11—C12—H12A	117.8	O7—C23—C24	117.3 (3)
C11—C12—H12B	117.8	C22—C23—C24	121.1 (3)
C11—C12—C13	59.4 (2)	C19—C24—C23	118.2 (3)
H12A—C12—H12B	115.0	C19—C24—H24	120.9
C13—C12—H12A	117.8	C23—C24—H24	120.9
C13—C12—H12B	117.8	H8A—O8—H8B	112 (5)
			
F1—C6—C7—N2	−2.4 (4)	C9—N1—C11—C13	−140.3 (3)
F1—C6—C7—C8	−178.8 (3)	C9—C4—C5—C6	0.7 (4)
O1—C1—C2—C3	−173.3 (3)	C10—N1—C9—C4	−3.6 (4)
O1—C1—C2—C10	4.3 (4)	C10—N1—C9—C8	175.5 (3)
O2—C1—C2—C3	5.9 (4)	C10—N1—C11—C12	117.4 (3)
O2—C1—C2—C10	−176.5 (3)	C10—N1—C11—C13	47.5 (4)
O3—C3—C4—C5	2.6 (4)	C10—C2—C3—O3	178.2 (3)
O3—C3—C4—C9	−179.1 (3)	C10—C2—C3—C4	−2.9 (4)
N1—C11—C12—C13	−106.9 (3)	C11—N1—C9—C4	−175.7 (2)
N1—C11—C13—C12	109.5 (3)	C11—N1—C9—C8	3.4 (4)
N2—C7—C8—C9	−175.5 (3)	C11—N1—C10—C2	175.1 (3)
N2—C14—C15—N3	−55.7 (3)	C14—N2—C7—C6	62.4 (4)
N3—C16—C17—N2	58.0 (3)	C14—N2—C7—C8	−121.4 (3)
C1—C2—C3—O3	−4.2 (4)	C14—N2—C17—C16	−59.9 (3)
C1—C2—C3—C4	174.7 (3)	C15—N3—C16—C17	−56.1 (3)
C1—C2—C10—N1	−177.1 (3)	C16—N3—C15—C14	54.5 (3)
C2—C3—C4—C5	−176.4 (3)	C17—N2—C7—C6	−166.4 (3)
C2—C3—C4—C9	2.0 (4)	C17—N2—C7—C8	9.8 (4)
C3—C2—C10—N1	0.5 (4)	C17—N2—C14—C15	59.2 (3)
C3—C4—C5—C6	179.1 (3)	O4—C18—C19—C20	−168.3 (3)
C3—C4—C9—N1	1.1 (4)	O4—C18—C19—C24	11.9 (4)
C3—C4—C9—C8	−178.0 (3)	O5—C18—C19—C20	11.1 (5)
C4—C5—C6—F1	178.0 (3)	O5—C18—C19—C24	−168.6 (3)
C4—C5—C6—C7	−1.1 (5)	O6—C21—C22—C23	−179.4 (3)
C5—C4—C9—N1	179.5 (3)	O7—C23—C24—C19	−177.1 (3)
C5—C4—C9—C8	0.4 (4)	C18—C19—C20—C21	178.9 (3)
C5—C6—C7—N2	176.7 (3)	C18—C19—C24—C23	179.2 (3)
C5—C6—C7—C8	0.3 (4)	C19—C20—C21—O6	−178.9 (3)
C6—C7—C8—C9	0.8 (4)	C19—C20—C21—C22	1.4 (4)
C7—N2—C14—C15	−167.8 (2)	C20—C19—C24—C23	−0.5 (4)
C7—N2—C17—C16	166.7 (2)	C20—C21—C22—C23	0.3 (4)
C7—C8—C9—N1	179.7 (3)	C21—C22—C23—O7	177.1 (3)
C7—C8—C9—C4	−1.2 (4)	C21—C22—C23—C24	−2.2 (4)
C9—N1—C10—C2	2.9 (4)	C22—C23—C24—C19	2.3 (4)
C9—N1—C11—C12	−70.4 (4)	C24—C19—C20—C21	−1.3 (4)

### 4-(3-Carboxy-1-cyclopropyl-6-fluoro-4-oxo-1,4-dihydroquinolin-7-yl)piperazin-1-ium chloride–3,5-hydroxybenzoic acid–water (1/1/1) (II) Hydrogen-bond geometry (Å, º)

**Table:**

*D*—H···*A*	*D*—H	H···*A*	*D*···*A*	*D*—H···*A*
O2—H2···O3	0.84	1.78	2.551 (3)	152
N3—H3*A*···O1^i^	0.91	1.75	2.652 (3)	172
N3—H3*B*···Cl1	0.91	2.30	3.106 (3)	148
C10—H10···F1^ii^	0.95	2.46	3.158 (4)	130
C12—H12*B*···O7^iii^	0.99	2.47	3.435 (4)	166
C14—H14*B*···F1	0.99	2.27	2.927 (3)	123
C16—H16*B*···Cl1^iv^	0.99	2.78	3.609 (3)	142
O4—H4···Cl1	0.84	2.28	3.082 (2)	160
O6—H6···Cl1^v^	0.84	2.40	3.232 (2)	170
O7—H7···O8	0.84	1.96	2.769 (3)	161
O8—H8*A*···Cl1^i^	0.88 (6)	2.51 (6)	3.362 (3)	164 (4)
O8—H8*B*···O5^vi^	0.82 (6)	2.05 (6)	2.865 (4)	170 (5)

Symmetry codes: (i) *x*, *y*+1, *z*−1; (ii) *x*, *y*−1, *z*; (iii) *x*−1, *y*−1, *z*+1; (iv) *x*−1, *y*, *z*; (v) *x*, *y*+1, *z*; (vi) *x*, *y*, *z*−1.
